# Comments on: Predictive factors for vaginal delivery in pregnant women with previous cesarean section: a case-control study

**DOI:** 10.61622/rbgo/2026rbgo108L23

**Published:** 2026-10-13

**Authors:** Çağdaş Nurettin Emeklioğlu

**Affiliations:** 1 Marmara University Pendik Training and Research Hospital Department of Obstetrics and Gynecology Division of Perinatology Istanbul Turkey Division of Perinatology, Department of Obstetrics and Gynecology, Marmara University Pendik Training and Research Hospital, Istanbul, Turkey

Dear Editor,

I have read with great interest the article by Reis et al. titled "Predictive factors for vaginal delivery in pregnant women with previous cesarean section: a case-control study" published in your esteemed journal.^(1)^ The authors present a very valuable and timely piece of work, identifying critical clinical, demographic, and obstetric predictors that favor a successful vaginal birth after cesarean (VBAC). Such studies are highly commendable and play an instrumental role in globally encouraging both clinicians and expectant mothers toward trial of labor after cesarean (TOLAC).^(2-4)^ This study holds significant relevance for countries like Turkey, where the rate of cesarean sections (C/S) has experienced an unprecedented and dramatic increase over the past decades. In regions with exceptionally high C/S rates, robust predictive models and clinical evidence are vital to build confidence among pregnant women and to dismantle the deep-rooted cultural and medical hesitations surrounding VBAC. In this context, I would like to offer some insights based on national healthcare strategies implemented in Turkey that could further optimize VBAC success rates globally when effectively integrated.

Firstly, the preparedness and psychological empowerment of the pregnant woman are paramount. In Turkey, the Ministry of Health has widely implemented standardized prenatal education facilities known as "Pregnancy Schools".^(5)^ These centers provide structured, comprehensive education regarding the physiological course of pregnancy, labor mechanisms, vaginal birth, pain coping strategies, and active labor positioning. Such educational spaces are immensely valuable; they demystify vaginal birth, alleviate maternal anxiety, and actively foster maternal motivation, which are essential precursors for a successful trial of labor.

Secondly, maternal motivation alone is insufficient without a highly supportive and secure clinical environment. To successfully increase VBAC rates, there is an urgent need to establish fully equipped, comprehensive tertiary healthcare facilities capable of managing sudden obstetric emergencies and potential catastrophic complications like uterine rupture. Such centers must house multi-disciplinary, highly experienced obstetric and neonatal teams operating 24/7. Alongside infrastructural readiness, systemic strategies and Professional guidelines must be formulated to legally and clinically encourage, support, and reassure obstetricians to confidently offer TOLAC to eligible candidates.

From a methodological perspective, the authors appropriately acknowledged that the interdelivery interval could not be evaluated because this information was inconsistently documented in the medical records. Nevertheless, this variable remains clinically relevant, as it has been associated with uterine rupture risk and may influence both TOLAC eligibility and the likelihood of successful VBAC. Future prospective studies incorporating complete interdelivery interval data, together with established predictors, may further improve the accuracy and generalizability of VBAC prediction models.

In conclusion, the study by Reis et al. represents an important contribution to the growing evidence supporting individualized TOLAC counseling. However, maximizing VBAC success requires more than accurate clinical prediction. Integrating structured prenatal education, ensuring institutional readiness, and prospectively evaluating additional clinically relevant variables may further enhance the implementation of VBAC strategies across diverse healthcare systems.

## Data Availability

The authors did not make the data from this article available in repositories prior to submission.
